# Supplementary material for: Clinical and cognitive improvement following full-spectrum, high-cannabidiol treatment for anxiety: open-label data from a two-stage, phase 2 clinical trial
Source: Commun Med (Lond). 2022 Nov 2;2:139. doi: 10.1038/s43856-022-00202-8 (PMC9628346; doi:10.1038/s43856-022-00202-8)
Supplement: Supplementary file 1 — Reporting Summary [file 43856_2022_202_MOESM1_ESM.pdf]

nature research

Corresponding author(s): Staci A. Gruber, Ph.D.

Last updated by author(s): Jan 19, 2022

Reporting Summary

Nature Research wishes to improve the reproducibility of the work that we publish. This form provides structure for consistency and transparency in reporting. For further information on Nature Research policies, see our Editorial Policies and the Editorial Policy Checklist.

Statistics

For all statistical analyses, confirm that the following items are present in the figure legend, table legend, main text, or Methods section.

n/a	Confirmed	
☐	☒	The exact sample size (*n*) for each experimental group/condition, given as a discrete number and unit of measurement
☐	☒	A statement on whether measurements were taken from distinct samples or whether the same sample was measured repeatedly
☐	☒	The statistical test(s) used AND whether they are one- or two-sided<br>*Only common tests should be described solely by name; describe more complex techniques in the Methods section.*
☒	☐	A description of all covariates tested
☐	☒	A description of any assumptions or corrections, such as tests of normality and adjustment for multiple comparisons
☐	☒	A full description of the statistical parameters including central tendency (e.g. means) or other basic estimates (e.g. regression coefficient) AND variation (e.g. standard deviation) or associated estimates of uncertainty (e.g. confidence intervals)
☐	☒	For null hypothesis testing, the test statistic (e.g. *F*, *t*, *r*) with confidence intervals, effect sizes, degrees of freedom and *P* value noted<br>*Give P values as exact values whenever suitable.*
☒	☐	For Bayesian analysis, information on the choice of priors and Markov chain Monte Carlo settings
☒	☐	For hierarchical and complex designs, identification of the appropriate level for tests and full reporting of outcomes
☐	☒	Estimates of effect sizes (e.g. Cohen's *d*, Pearson's *r*), indicating how they were calculated

Our web collection on statistics for biologists contains articles on many of the points above.

Software and code

Policy information about availability of computer code

Data collection	Data were collected via REDCap.
Data analysis	Data were analyses using SPSS (v.24).

For manuscripts utilizing custom algorithms or software that are central to the research but not yet described in published literature, software must be made available to editors and reviewers. We strongly encourage code deposition in a community repository (e.g. GitHub). See the Nature Research guidelines for submitting code & software for further information.

Data

Policy information about availability of data

All manuscripts must include a data availability statement. This statement should provide the following information, where applicable:

- Accession codes, unique identifiers, or web links for publicly available datasets
- A list of figures that have associated raw data
- A description of any restrictions on data availability

The datasets generated during and/or analyzed during the current study are available from the corresponding author on reasonable request.

Field-specific reporting

Please select the one below that is the best fit for your research. If you are not sure, read the appropriate sections before making your selection.

☐ Life sciences ☒ Behavioural & social sciences ☐ Ecological, evolutionary & environmental sciences

For a reference copy of the document with all sections, see nature.com/documents/nr-reporting-summary-flat.pdf

Behavioural & social sciences study design

All studies must disclose on these points even when the disclosure is negative.

Study description	The open-label phase of clinical trial (ClinicalTrials.gov: NCT02548559) assessed four-weeks of treatment with a full-spectrum, high-cannabidiol (CBD) sublingual solution in patients with moderate-to-severe anxiety.
Research sample	Fourteen patients with moderate-to-severe anxiety defined as ≥16 on the Beck Anxiety Inventory (BAI) or ≥11 on the Overall Anxiety Severity and Impairment Scale (OASIS).
Sampling strategy	Patients were recruited from the New England area through online advertisements. Enrollment for the open-label phase was originally planned to be capped at 16 participants to determine dosing and tolerability; however, the onset of the COVID-19 resulted in early closure to enrollment with complete data on 14 patients. Power analyses indicated that shifting the sample size from 16 to 14 completed patients only slightly impacted the required effect size ($\eta 2$=.11 vs $\eta 2$=.12).
Data collection	The primary outcome variables to assess anxiety were the Beck Anxiety Inventory (BAI), Overall Anxiety Severity and Impairment Scale (OASIS), State-Trait Anxiety Inventory (STAI), and Hamilton Anxiety Rating Scale (HAM-A). Patients also completed the following assessments of mood, sleep disturbance, sexual function, and quality of life: Beck Depression Inventory (BDI), Profile of Mood States (POMS), Positive and Negative Affect Schedule (PANAS), Beck Hopelessness Scale (BHS), Beck Scale for Suicide Ideation (BSS), Pittsburgh Sleep Quality Index (PSQI), Arizona Sexual Experience Scale (ASEX), and the Medical Outcomes Survey Short Form-36 (SF-36). Executive function was assessed using the Stroop Color Word Test, Trail Making Test (TMT), Wisconsin Card Sorting Task (WCST), Multi-Source Interference Task (MSIT), Letter-Number Sequencing test (LNS), Digit Symbol Substitution Task (DSST), and Controlled Oral Word Association Test (COWAT). Visual memory was assessed using the Benton Visual Retention Task (BVRT) and verbal memory via the Rey Auditory Verbal Learning Task (RAVLT).
Timing	Data were collected June 20, 2018-Feb 12, 2020.
Data exclusions	One patient was discontinued and excluded from analyses for use of another cannabinoid product during the trial.
Non-participation	223 people were assessed for eligibility, out of which 182 did not meet inclusion criteria, 16 were lost to follow-up/no-showed before enrollment, 6 declined to participate, and 4 were moved to the double-blind phase due to COVID-related delays. In total, 15 patients enrolled in the study and received the allocated intervention; 14 completed the entire study.
Randomization	Not applicable. Open-label phase.

Reporting for specific materials, systems and methods

We require information from authors about some types of materials, experimental systems and methods used in many studies. Here, indicate whether each material, system or method listed is relevant to your study. If you are not sure if a list item applies to your research, read the appropriate section before selecting a response.

Materials & experimental systems

n/a	Involved in the study	
☒	☐	Antibodies
☒	☐	Eukaryotic cell lines
☒	☐	Palaeontology and archaeology
☒	☐	Animals and other organisms
☐	☒	Human research participants
☐	☒	Clinical data
☒	☐	Dual use research of concern

Methods

n/a	Involved in the study	
☒	☐	ChIP-seq
☒	☐	Flow cytometry
☒	☐	MRI-based neuroimaging

Human research participants

Policy information about studies involving human research participants

Population characteristics	This sample was comprised of fourteen patients (11F, 3M) aged 41.36 ± 16.89 with moderate-to-severe anxiety defined as

Population characteristics	≥16 on the Beck Anxiety Inventory (BAI) or ≥11 on the Overall Anxiety Severity and Impairment Scale (OASIS). Baseline averages on these anxiety scales were BAI=20.29 ± 9.92 and OASIS=11.29 ± 1.49.
Recruitment	Patients were recruited from the New England area through online advertisements. Standard clinical thresholds of anxiety were used in order to ensure patients met the inclusion criteria of moderate-to-severe anxiety (≥16 on the Beck Anxiety Inventory [BAI] or ≥11 on the Overall Anxiety Severity and Impairment Scale [OASIS]).
Ethics oversight	This study was approved by the Mass General Brigham Institutional Review Board and carried out in accordance with the Declaration of Helsinki.

Note that full information on the approval of the study protocol must also be provided in the manuscript.

Clinical data

Policy information about clinical studies

All manuscripts should comply with the ICMJE guidelines for publication of clinical research and a completed CONSORT checklist must be included with all submissions.

Clinical trial registration	ClinicalTrials.gov: NCT02548559
Study protocol	https://clinicaltrials.gov/ct2/show/NCT02548559
Data collection	Data were collected June 20, 2018-Feb 12, 2020 at McLean Hospital, Belmont, MA.
Outcomes	The primary outcome variables assessed anxiety: Beck Anxiety Inventory (BAI), Overall Anxiety Severity and Impairment Scale (OASIS), State-Trait Anxiety Inventory (STAI), and Hamilton Anxiety Rating Scale (HAM-A). Secondary outcome variables assessed mood, sleep disturbance, sexual function, and quality of life: Beck Depression Inventory (BDI), Profile of Mood States (POMS), Positive and Negative Affect Schedule (PANAS), Beck Hopelessness Scale (BHS), Beck Scale for Suicide Ideation (BSS), Pittsburgh Sleep Quality Index (PSQI), Arizona Sexual Experience Scale (ASEX), and the Medical Outcomes Survey Short Form-36 (SF-36). Additional secondary outcome variables assessed cognition. Executive function was assessed using the Stroop Color Word Test, Trail Making Test (TMT), Wisconsin Card Sorting Task (WCST), Multi-Source Interference Task (MSIT), Letter-Number Sequencing test (LNS), Digit Symbol Substitution Task (DSST), and Controlled Oral Word Association Test (COWAT). Visual memory was assessed using the Benton Visual Retention Task (BVRT) and verbal memory via the Rey Auditory Verbal Learning Task (RAVLT).
