# Supplementary material for: Clinical and cognitive improvement following full-spectrum, high-cannabidiol treatment for anxiety: open-label data from a two-stage, phase 2 clinical trial
Source: Commun Med (Lond). 2022 Nov 2;2:139. doi: 10.1038/s43856-022-00202-8 (PMC9628346; doi:10.1038/s43856-022-00202-8)
Supplement: Supplementary file 2 — Supplemental Material [file 43856_2022_202_MOESM2_ESM.pdf]

Clinical and Cognitive Improvement Following Full-Spectrum, High-Cannabidiol Treatment for Anxiety: An Open-Label, Phase 2 Trial

Supplemental Materials:

Supplemental Table 1. Changes in Mood, Sleep Disturbance, Sexual Dysfunction, and Quality of Life Over the Course of 4 Weeks of Treatment with a High-Cannabidiol (CBD) Sublingual Study Product: Autoregressive Linear Mixed Models (Two-Tailed)

Supplemental Table 2. Pearson Correlations Assessing the Association Between Positive and Negative Expectancies and the Difference Score (Baseline minus Week 4) of Clinical Outcome Variables

Supplemental Table 1. Changes in Mood, Sleep Disturbance, Sexual Dysfunction, and Quality of Life Over the Course of 4 Weeks of Treatment with a High-Cannabidiol (CBD) Sublingual Study Product: Autoregressive Linear Mixed Models (Two-Tailed)

Clinical Scale	Mixed Model	Baseline *n*=14 (ref.)	Week 1 *n*=14	Week 2 *n*=14	Week 3 *n*=14	Week 4 *n*=14	Baseline to Week 4
	F p (η_p^2)	Mean [95% CI]	Estimate (*p*) [95% CI]	Estimate (*p*) [95% CI]	Estimate (*p*) [95% CI]	Estimate (*p*) [95% CI]	Percent Change
**Depression Scales**							
Beck Depression Inventory (BDI)	**15.72 <.001 (.65)**	20.07 [16.56, 23.58]	**-8.43 (<.001) [-11.28, -5.57]**	**-11.93 (<.001) [-15.59, -8.27]**	**-13.57 (<.001) [-17.68, -9.46]**	**-15.64 (<.001) [-20.03, -11.25]**	**-77.94%**
Beck Hopelessness Scale (BHS)	**16.06 .001 (.55)**	8.29 [5.83, 10.74]	-	-	-	**-5.36 (.001) [-8.25, -2.47]**	**-64.66%**
Beck Scale for Suicide Ideation (BSS)	3.06 .104 (.19)	0.64 [-0.29, 1.57]	-	-	-	-0.29 (.104) [-0.64, 0.07]	-44.44%
**Profile of Mood States (POMS)**							
Total Mood Disturbance	**10.95 <.001 (.63)**	65.86 [51.02, 80.69]	**-26.57 (<.001) [-36.05, -17.09]**	**-36.14 (<.001) [-48.76, -23.52]**	**-34.93 (<.001) [-49.54, -20.31]**	**-41.93 (<.001) [-57.95, -25.91]**	**-63.67%**
**Positive and Negative Affect Schedule (PANAS)**							
Positive Affect	1.88 .129 (.14)	29.36 [25.18, 33.53]	-0.36 (.844) [-3.98, 3.27]	-2.21 (.338) [-6.80, 2.37]	-1.29 (.616) [-6.38, 3.81]	3.21 (.238) [-2.18, 8.60]	10.95%
Negative Affect	**7.49 <.001 (.44)**	26.21 [22.41, 30.02]	**-8.00 (.001) [-12.72, -3.28]**	**-12.29 (<.001) [-17.51, -7.07]**	**-11.57 (<.001) [-16.91, -6.23]**	**-12.71 (<.001) [-18.09, -7.34]**	**-48.50%**
**Sleep Disturbance**							
Pittsburgh Sleep Quality Index (PSQI)	**20.27 .001 (.61)**	9.21 [7.57, 10.86]	-	-	-	**-3.36 (.001) [-4.97, -1.75]**	**-36.43%**
**Sexual Dysfunction**							
Arizona Sexual Experiences Scale (ASEX)[a]	1.55 .237 (.11)	15.08 [12.03, 18.12]	-	-	-	-0.54 (.237) [-1.48, 0.40]	-3.57%
**Medical Outcomes Survey Short Form (SF-36)**							
Physical Function	4.10 .064 (.24)	78.57 [66.17, 90.97]	-	-	-	5.36 (.064) [-0.36, 11.07]	6.82%
Role Limitations: Physical Health	2.58 .133 (.17)	60.71 [39.51, 81.91]	-	-	-	16.07 (.133) [-5.57, 37.71]	26.47%
Role Limitations: Emotional Problems	**32.50 <.001 (.71)**	26.19 [7.33, 45.05]	-	-	-	**47.62 (<.001) [29.57, 65.67]**	**181.82%**
Energy/Fatigue	**8.50 .012 (.40)**	30.36 [18.54, 42.18]	-	-	-	**16.79 (.012) [4.35, 29.22]**	**55.29%**
Emotional Well-Being	**56.52 <.001 (.81)**	49.14 [42.10, 56.19]	-	-	-	**24.86 (<.001) [17.71, 32.00]**	**50.58%**
Social Function	**37.16 <.001 (.74)**	52.68 [41.57, 63.79]	-	-	-	**29.46 (<.001) [19.02, 39.91]**	**55.93%**
Pain	4.74 .049 (.27)	59.82 [45.53, 74.12]	-	-	-	10.18 (.049) [0.07, 20.28]	17.01%
General Health	2.98 .108 (.19)	55.00 [45.93, 64.07]	-	-	-	6.43 (.108) [-1.61, 14.47]	11.69%

Bold numbers are significant at $p \leq .010$ for 5 timepoints and $p \leq .025$ for 2 timepoints.
Significance is only noted for estimates relative to the baseline reference group.
Note: For the BDI, BHS, BSS, POMS, PANAS negative affect, PSQI, and ASEX scales, reduced estimates indicate clinical improvement. For the PANAS positive affect, and SF-36 scales, increased estimates indicate clinical improvement.
[a] *n*=13: one patient was not sexually active and therefore did not complete the ASEX

Supplemental Table 2. Pearson Correlations Assessing the Association Between Positive and Negative Expectancies and the Difference Score (Baseline minus Week 4) of Clinical Outcome Variables

Clinical Outcome Variables	Marijuana Effect Expectancy Questionnaire-Brief (MEEQ-B)	
	Positive Expectancies	Negative Expectancies
**Anxiety Scales**		
Beck Anxiety Inventory (BAI)	*r*=.048 *p*=.871	*r*=-.185 *p*=.526
Overall Anxiety Severity and Impairment Scale (OASIS)	*r*=-.377 *p*=.184	*r*=-.056 *p*=.849
Hamilton Anxiety Rating Scale (HAM-A)	*r*=-.002 *p*=.994	*r*=-.086 *p*=.770
State-Trait Anxiety Inventory (STAI): State	*r*=-.125 *p*=.670	*r*=.137 *p*=.641
State-Trait Anxiety Inventory (STAI): Trait	*r*=-.161 *p*=.583	*r*=.100 *p*=.735
**Depression Scales**		
Beck Depression Inventory (BDI)	*r*=-.067 *p*=.820	*r*=.043 *p*=.883
Beck Hopelessness Scale (BHS)	*r*=-.193 *p*=.509	*r*=.265 *p*=.360
Beck Scale for Suicide Ideation (BSS)	*r*=.283 *p*=.328	*r*=-.274 *p*=.343
**Profile of Mood States (POMS)**		
Total Mood Disturbance	*r*=-.267 *p*=.356	*r*=.186 *p*=.524
**Positive and Negative Affect Schedule (PANAS)**		
Positive Affect	*r*=.148 *p*=.613	*r*=-.352 *p*=.217
Negative Affect	*r*=-.017 *p*=.953	*r*=-.168 *p*=.566
**Sleep Disturbance**		
Pittsburgh Sleep Quality Index (PSQI)	*r*=.136 *p*=.644	*r*=.177 *p*=.545
**Sexual Dysfunction**		
Arizona Sexual Experiences Scale (ASEX)[a]	*r*=-.432 *p*=.140	*r*=.625 *p*=.022
**Medical Outcomes Survey Short Form (SF-36)**		
Physical Function	*r*=.400 *p*=.157	*r*=-.426 *p*=.128
Role Limitations: Physical Health	*r*=.563 *p*=.036	*r*=-.282 *p*=.329
Role Limitations: Emotional Problems	*r*=.371 *p*=.191	*r*=-.148 *p*=.615
Energy/Fatigue	*r*=.501 *p*=.068	*r*=-.056 *p*=.849
Emotional Well-Being	*r*=.083 *p*=.779	*r*=.014 *p*=.962
Social Function	*r*=.227 *p*=.436	*r*=.079 *p*=.788
Pain	*r*=.191 *p*=.514	*r*=.224 *p*=.442
General Health	*r*=.429 *p*=.126	*r*=-.437 *p*=.118

Unless otherwise noted, n=14

Bold numbers are significant at $p \leq .010$

[a] *n*=13: one patient was not sexually active and therefore did not complete the ASEX
