# Supplementary material for: Clinical and cognitive improvement following full-spectrum, high-cannabidiol treatment for anxiety: open-label data from a two-stage, phase 2 clinical trial
Source: Commun Med (Lond). 2022 Nov 2;2:139. doi: 10.1038/s43856-022-00202-8 (PMC9628346; doi:10.1038/s43856-022-00202-8)
Supplement: Supplementary file 4 — Description of Additional Supplementary Files [file 43856_2022_202_MOESM4_ESM.pdf]

Description of Additional Supplementary Files

File Name: Supplementary Data 1

Description: Source Data for Primary Outcomes
